# Letter to the Editor: Offloading diabetic wounds

**DOI:** 10.1002/jfa2.70018

**Published:** 2024-11-25

**Authors:** Mary Holbrook‐Martin, Sarah Curran

**Affiliations:** ^1^ Tissue Health Plus Huntington WV USA

Dear Editor,

The article by Ting et al. [1] provides valuable insights into the use of moldable offloading devices for diabetic foot ulcers. Suitable options for a variety of patients are always beneficial. However, there are several areas where further research and clarification would be helpful to enhance our understanding and improve patient care.

Firstly, the lack of specific information regarding wound treatments and dressings applied under the fiberglass back slab device is a significant omission. This information is crucial as it could potentially influence wound healing outcomes. Future studies should meticulously document and report on the types of dressings and treatments used in conjunction with the offloading device.

Secondly, while the authors state that the device has been successful in offloading for their particular group of patients, the absence of quantitative data to support this claim is noteworthy. To substantiate the evidence of the device, it would be beneficial to conduct studies that include objective measurements of pressure reduction. This could involve the use of pressure mapping systems or other validated tools to quantify the offloading achieved by the device.

Integration of remote monitoring technologies presents an exciting opportunity for advancement in this field [2]. These smart devices, capable of continuous pressure monitoring, could provide valuable real‐time data on the effectiveness of offloading interventions [3]. Incorporating such technologies into future studies of moldable offloading devices could yield more comprehensive and accurate assessments of their performance.

Furthermore, it would be beneficial to conduct comparative studies that evaluate the moldable offloading device against other established offloading methods using the same technology. This would help contextualize its effectiveness and potentially identify specific patient populations for which it may be particularly advantageous.

In the realm of diabetic foot care, where effective offloading is paramount to successful wound healing, continued research and innovation are essential. While the work of Ting et al. [1] represents a step forward, there remains ample opportunity for further investigation and refinement of offloading techniques. By addressing these areas in future research, we can work toward developing more effective, evidence‐based approaches to managing diabetic foot ulcers, ultimately improving patient outcomes and quality of life.
